# Qualitative evaluation of a form for standardized information exchange between orthopedic surgeons and occupational physicians

**DOI:** 10.1186/1472-6963-6-144

**Published:** 2006-11-02

**Authors:** Elske Faber, Alex Burdorf, Anne Loes van Staa, Harald S Miedema, Jan AN Verhaar

**Affiliations:** 1 Netherlands Expert Centre for Workrelated Musculoskeletal Disorders, Erasmus MC, Rotterdam, The Netherlands; 2 Public Health, Erasmus MC, Rotterdam, The Netherlands; 3 Institute for Health Policy and Management, Erasmus MC, Rotterdam, The Netherlands; 4 Orthopedics, Erasmus MC, Rotterdam, The Netherlands

## Abstract

**Background:**

Both occupational physicians and orthopedic surgeons can be involved in the management of work relevant musculoskeletal disorders. These physicians hardly communicate with each other and this might lead to different advices to the patient. Therefore, we evaluated a standardized information exchange form for the exchange of relevant information between the orthopedic surgeon and the occupational physician. The main goals of this qualitative study are to evaluate whether the form improved information exchange, whether the form gave relevant information, and to generate ideas to further improve this information exchange.

**Methods:**

The information exchange form was developed in two consensus meetings with five orthopedic surgeons and five occupational physicians. To evaluate the information exchange form, a qualitative evaluation was set up. Structured telephone interviews were undertaken with the patients, interviews with the physicians were face-to-face and semi-structured, based on a topic list. These interviews were recorded and literally transcribed. Each interview was analyzed separately in Atlas-Ti.

**Results:**

The form was used for 8 patients, 7 patients agreed to participate in the qualitative evaluation. All three orthopedic surgeons involved and three of the six involved occupational physicians agreed to be interviewed. The form was transferred to 4 occupational physicians, the other 3 patients recovered before they visited the occupational physician. The information on the form was regarded to be useful. All orthopedic surgeons agreed that the occupational physician should take the initiative. Most physicians felt that the form should not be filled out for each patient visiting an orthopedic surgeon, but only for those patients who do not recover as expected. Orthopedic surgeons suggested that a copy of the medical information provided to the general practitioner could also be provided to occupational physicians.

**Conclusion:**

The information exchange form was regarded to be useful and could be used in practice. The occupational physician should take the initiative for using this form and most physicians felt the information should only be exchanged for patients who do not recover as expected. That means that the advantage of giving information early in the treatment is lost.

## Background

Several physicians may be involved in the management of work relevant musculoskeletal disorders in the Netherlands; these are the general practitioner, the occupational physician and sometimes a medical specialist. A worker with musculoskeletal complaints usually starts care seeking by a visit to his general practitioner (GP). The GP is responsible for diagnosis and treatment and may refer to a medical specialist. In the Dutch health care system every employee has also access to occupational health care. The occupational physician usually becomes involved when a worker is on sick leave and will advise on necessary adaptations in work or at the workplace. Hence, a patient may receive advice from several physicians for the same health problem. These advices can differ from each other or even conflict with each other, since the medical specialist and the occupational health physician have different goals and advise the patient on different aspects of the musculoskeletal disorder.

Different or conflicting advices might lead to a prolonged duration of sick leave. Several studies [1-3] indicate that visiting a medical specialist is associated with a longer duration of sickness absence, even after adjustment for nature and severity of the musculoskeletal complaint. In another study occupational physicians reported that they felt that treatment by a general practitioner or medical specialist sometimes was an obstacle for return to work [4]. It is believed that better collaboration and better information exchange between physicians may limit long-term sick leave [5-7].

In 2000 a study showed that there is little communication between medical specialists (amongst others orthopedic surgeons) and occupational physicians. When communication took place, it was usually the occupational physician initiating the contact and most of the time it concerned an information request by mail. Although more than 80% of the participating orthopedic surgeons reported that they wanted to improve their collaboration with occupational physicians, it proved to be difficult in practice [8]. Hence, we designed an intervention to facilitate communication and to overcome some of the known barriers. The barriers involved in interdisciplinary collaboration range from not knowing how to reach each other to not finding the other party an equal collaboration-partner [9-12]. To improve information exchange and collaboration, it should be made as easy as possible, possibly even with standard guidelines [6,7,13]. The importance of administrative formalization initiatives has been stressed as an essential tool to enhance collaboration [14].

This study consists of two parts; first we developed a standardized information exchange form for the exchange of relevant information between the orthopedic surgeon and the occupational physician, in order to facilitate the latter in return to work management. The practical use of this form was evaluated in a qualitative study. The goal was to evaluate whether the patients and physicians appreciated the information exchange by means of the form, whether the form provided relevant information to the occupational physicians, and to generate ideas to further improve this information exchange.

## Methods

### Development of communication form

The information exchange form was developed in two consensus meetings with five orthopedic surgeons and five occupational physicians. We developed three versions of the form, related to frequent disorders with an established impact on sick leave: non-specific low back pain, impingement syndrome of the shoulder, and meniscal tears and knee ligament injuries [see additional file 1]. The three forms are equal in the kind of information they provide, but the details are specific for the injured part of the body.

In the information exchange form the following information is provided: contact information of the orthopedic surgeon, general information about the patient, the preliminary diagnosis, the proposed trajectory (additional diagnostics and therapy), current functional limitations, and provisional prognosis on recovery. The functional limitations section was based on a list used by insurance physicians to decide whether a patient is entitled to receive a work disability pension [15]. The participating physicians agreed on the fact that the information exchange form should be filled out as soon as possible in the treatment trajectory. The short-term disabilities and preliminary diagnosis should be given early in the treatment trajectory, e.g. the first or second consultation.

The information exchange form complies with the regulations of the Royal Dutch Medical Association for exchange of information between curative care and occupational health care. This entails that the patient always has to provide written consent for the information that will be exchanged. The form asks for the signature of the patient that he was informed appropriately and agrees with the exchange of the information on the form. All participants agreed with the patient being the information carrier, since the patient visits both the orthopedic surgeon and the occupational physician and this provides an easy way of reaching each other.

### Evaluation of the information exchange form

The feasibility of the information exchange form was evaluated in a qualitative study. Originally a controlled trial was set up with ten orthopedic surgeons using the information exchange form and ten orthopedic surgeons giving care as usual. The orthopedic surgeons informed new patients who were referred by a general practitioner for a first consult for knee (meniscal tears, ACL), shoulder (impingement) or non-specific back pain about the project. Only patients who had a paid occupation, were on sick leave or had a high risk for sick leave, and required treatment were included. Exclusion criteria were severe co-morbidity, sick leave due to another cause, arthritis, and practicing top sport (national competition). Patients willing to participate were asked to return the informed consent form prior to their next visit to the OS. This trial was in compliance with the Helsinki Declaration and was approved by the medical ethical committee of Erasmus MC.

After an inclusion period of 9 months, the information exchange form was used for only 8 patients, therefore a quantitative evaluation was not feasible. To evaluate the use of the information exchange form, a qualitative evaluation was set up. All included patients and their orthopedic surgeon and occupational physician, were asked to participate in an interview. The medical ethical committee of Erasmus MC approved with the interviews with physicians and patients in addition to the larger trial.

Structured telephone interviews with each of the patients were undertaken lasting approximately 15 minutes and the answers given by the patients were written down. Each patient was asked the same questions. The interviews with each physician were face-to-face and semi-structured, based on a topic list. These interviews lasted approximately 45 minutes and were recorded and literally transcribed. All interviews took place in the fall of 2005. Table 1 shows a summary of the questions and topics in the interviews.

Each interview was analyzed separately in Atlas-Ti. The analysis was primarily based on the topics from the topic lists. EF and AS did the analysis of the interviews. First, all data were coded and based on these codes the information was structured and analyzed [16]. A member check was performed by sending this manuscript for approval to the participating physicians.

## Results

**Figure 1:**
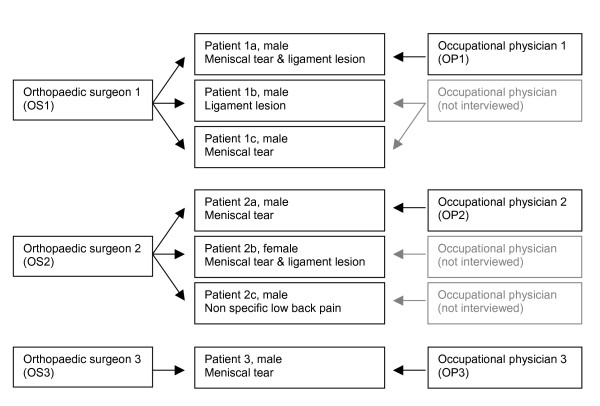
Participants.

The form was used for only 8 patients by three orthopedic surgeons. The other 7 orthopedic surgeons did not include patients in the study and, therefore, did not use the form. Of 8 patients, 7 agreed to participate in the qualitative evaluation. These 7 patients were treated by 3 orthopedic surgeons and 6 occupational physicians. All three orthopedic surgeons and three occupational physicians agreed to be interviewed. Figure 1 is a diagram in which all participants are schematically represented. Most patients had knee disorders and one patient had non-specific low back pain.

### Did the form improve information exchange?

Of the 7 patients in this evaluation, all remembered that their orthopedic surgeon mentioned or filled out the form. However, three forms did not reach the occupational physician. Two patients (1c, 2b) had not visited their occupational physician and also not mailed the form. Patient 3 said that his orthopedic surgeon had sent the form to his occupational physician, however, the occupational physician had not received the form. In all cases where the occupational physician had not received the form, the patient was recovered before a consultation with the occupational physician was planned. Four forms were given or mailed to the occupational physician; patient 2c had send it over mail, even though he did not have an appointment with his occupational physician, and the other three patients handed the form over to their occupational physician.

The form was given to two of the three interviewed occupational physicians. The occupational physician not receiving the form was the one referring his patient to another orthopedic surgeon for second opinion. His opinion on the form was ambiguous: *"The form passes the role of the occupational physician with regard to the functional limitations. However, my thoughts are ambiguous since I have just said that when I am unsure about the functional limitations, that is just the information I need from the orthopedic surgeon"(OP3)*. The two occupational physicians who had received the form answered that it gave them enough information to plan the patient's rehabilitation to work: *"It was, for me, a guide to plan the work rehabilitation relatively fast and easy"(OP1)*.

Patient 2a answered that he felt that the form had resulted in better communication, since the occupational physician now knew which orthopedic surgeon to contact. His occupational physician referred patient 3 to another orthopedic surgeon; in this case the occupational physician did not need information from the first orthopedic surgeon and had not received the form either. Patients 1a and 1b assumed that the form was read, but did not know whether it resulted in anything else.

None of the orthopedic surgeons remembered to be contacted by the occupational physicians for additional information. Since only a few patients per orthopedic surgeon were included, they could not answer the question whether the form improved information exchange.

### Did the information exchange form provide relevant information?

The three orthopedic surgeons considered the forms to be complete and useful. They had no difficulties filling out the form, and all answered that the five minutes necessary to fill it out was a reasonable amount of time. Two of them had instructed their secretaries to inform the patients about the form and the research in order to save time during the consultation. They felt that the information exchange form asked similar information as usually asked by occupational physicians.

The forms gave the occupational physicians information on functional limitations, which helped them to help the patient return to work: *"A clinician gives clinical, health related information on specific functional limitations*. <...> *That gives an estimation, when an orthopedic surgeon can give this information that is important information. It takes questions away" (OP2)*. Besides the information provided on the form, there was another reason that made it useful: *"It gives you the opportunity to contact the treating specialist" (OP3)*.

Two occupational physicians wanted to add information to the form. One occupational physician (OP3) said that the form might be too strict; he answered that it should provide room for extra information or explanation. Another occupational physician (OP2) felt that information about a patient's medical history and how he recovered from possible earlier treatments was missing. The third occupational physician found the information on the form complete: *"It is more than I would have expected. Usually, when I ask similar questions I do not receive the answers this complete. Especially not regarding the functional limitations. May be it is so easy, because all the orthopedic surgeon needs to do is to put the crosses in the right squares"(OP1)*.

One of the orthopedic surgeons did not want to fill out the part on functional limitations: *"Once it is on paper, it is regarded as a fact. ... Also I do not know where the patient works and what his job is. To me that is part of the job of an occupational physician. My predecessor always told me: you have to be able to defend everything you write down" (OS3)*.

When asked whether the use of the form made orthopedic surgeons more aware of the fact that a patient also has a role as a worker, only one of them agreed: *"You are more aware of the fact that the patient also has a function in life" (OS2)*. However, all three surgeons said that they usually asked their patients about their job. Asking questions about a patient's job does not mean that they also inform the patient on their functional limitations at work. The participating surgeons only discussed functional limitations when the patient asks for information on what he can and cannot do. Two of the surgeons preferred not to give direct information about consequences for the patient's work: *"Yes, when they ask for it. In activities of daily live. Never for their work, and that is because I do not know the company and workplace"(OS1)*.

### Ideas to further improve this information exchange

All participants, both physicians and patients, agreed with the patient being the information carrier. One orthopedic surgeon said: *"There is no reason, for me, to keep the information on the forms secret for the patient. He is allowed to see all information in his medical file, including this information"(OS1)*. Both occupational physicians and most orthopedic surgeons felt that the patient would take better care of the forms than when it is sent with regular mail. However, one orthopedic surgeon questioned whether the forms would reach the occupational physician. He had no objections against giving the form to the patient, but would also send it separately to the occupational physician.

All interviewed physicians would not mind using the form in future, as one of the occupational physicians said: *"It gives you the possibility to contact each other" (OP3)*. In this study the orthopedic surgeon took the initiative to inform the occupational physician. However, all orthopedic surgeons said that the occupational physician should take the initiative since it is their responsibility to manage the patient's work rehabilitation: *"I think that the occupational physician should take the initiative, be more active. That is his work. Our work is to cure people. And we have nothing to do with the fact whether this man works or not"(OS1)*.

Another option mentioned was that the occupational physician could ask for a copy of the letter written to the general practitioner, with medical information on the diagnosis and treatment instead of using this form. *"The letter to the general practitioner is a moment when you already exchange information. So if you can limit information exchange to one moment it is no extra effort" (OS2)*.

All orthopedic surgeons and 2 occupational physicians felt that the form should not be filled out for each patient visiting an orthopedic surgeon, but only for those patients who do not recover as expected. *"In cases with chronic musculoskeletal complaints or when there is a complication in the recovery" (OP2)*.

## Discussion

The results show that the information on the information exchange form was regarded to be useful. Two participating occupational physicians stated that it was useful and that it helped them to plan the reintegration to work. The orthopedic surgeons answered that the information provided through the forms could be useful for the occupational physicians. However, the form was hardly used by the participating orthopedic surgeons since only 8 patients were included. Of these patients, only 4 gave the form to their occupational physician and one patient answered that it had resulted in better communication. The fact that the form was only used for 8 patients can have several reasons. One possible reason is that the inclusion criteria for the study were too strict. However, in an additional survey among new patients visiting an orthopedic outpatient clinic we have estimated that approximately 4% of all new patients matched the inclusion criteria. This gives reason to believe that our inclusion criteria were not too strict. Other reasons can be lack of time, or the fact that the form had to be filled out before the treatment had taken place. Also, orthopedic surgeons might not see work as an important factor to take into consideration for their treatment; they treat the disorder and advice the patient on functional limitations in general.

It was decided that the form should be filled out early in the treatment trajectory since it was expected that patients would have had their complaints for a longer period already and early intervention can help a worker to return to work faster. This meant that the orthopedic surgeon filled out the form without an information request from the occupational physician, in the same way as the letter they normally send to the general practitioner. The participating orthopedic surgeons stated that the occupational physicians should take the initiative for the use of the form and both orthopedic surgeons and occupational physicians felt that it should only be used in those cases where the patient does not recover as expected. This would save time and occupational physicians will usually only ask for information when recovery does not work out as expected. Hence, the structured form may be used better at a later stage in the treatment trajectory and limited to those patients where it becomes clear that recovery will be delayed. The disadvantage of this timing may be that for some patients the orthopedic surgeon no longer is in charge of the treatment.

The fact that the patient was the carrier of the information was seen as a good and effective way to reach the colleague-physician. Since 2002 a new law has been implemented in the Netherlands, the Gatekeeper Improvement Act (Wet Verbetering Poortwachter), giving responsibility for the duration of sick leave not only to the employer and occupational physician, but also to the employee on sick leave. In this study we gave the patient the responsibility to transfer the information exchange form to the occupational physician, and thereby to transfer medical information on the disorder. In this study, only four forms were given or sent to the occupational physician. The patients not transferring the form to their occupational physician recovered before their first visit to the occupational physician was planned and, thus, the information on the form was not needed to plan the rehabilitation. We have no indication that patients would object against the transfer of medical information from the specialist to the occupational physician. We do not think it is an important barrier in most cases, since in the before mentioned Act patients have the obligation and responsibility to fully cooperate with regard to return to work. Most patients are motivated to support all actions that are necessary for that, including information transfer to the occupational physician. However, a minority of patients could be reluctant to give permission for information transfer to the occupational physician, because they are afraid this information will be given to the employer. Although this is forbidden under Dutch privacy and physician-patient legislation, this fear is sometimes present and is enhanced by the fact that the employer pays for the work of the occupational physician directly or indirectly (via an occupational health service).

In the Dutch health care system the tasks and responsibilities of curative health care and occupational health care are strictly divided. Curative health care providers advice on and give medical treatment and occupational health care providers manage work rehabilitation. An occupational physician is an expert in translating functional limitations to limitations and possibilities at work. The main goal of the information exchange form was to inform the occupational physician on the diagnosis, treatment and functional limitations from a medical point of view. Due to the fact that the occupational physician is responsible for work rehabilitation, the information exchange form was directed to convey information from the orthopedic surgeon to the occupational physician. In other health care systems clinical health care providers can have the responsibility for return to work or the decision that a patient is fit for work. In those cases the information exchange might be directed both to and from the occupational health care in order to provide all parties involved in the management of the disorder and sick leave with necessary information.

Many patients visiting an orthopedic surgeon ask for information about their limitations in daily life related to the diagnosis and prognosis. Work is part of the daily life activities of many patients. However, most orthopedic surgeons, just as general practitioners, are not trained in occupational health [17,18]. They might perceive difficulties when asked for advice on the workability of a patient, without knowing the specific capabilities required to work in a specific work situation. In this study, one of the orthopedic surgeons did not want to give information on functional limitations due to fear of possible legal consequences. However, Dasinger [19] showed that workers with a worker's compensation claim for low back injury are more likely to get off disability-benefit status when they were informed on their readiness to return to work by their treating physician. Early intervention by a treating physician can help a worker to resist the negative effects of a system that discourages early return to work [20,21]. Since orthopedic surgeons treat a disorder and do not usually seem to consider work as part of this treatment, this may hamper collaboration.

The question remains whether using this form can improve information exchange. The form was only used for eight patients in this study, of which only four transferred it to their occupational physician. The form is easy to fill out for the orthopedic surgeon and provides the occupational physician with medical information, planning of treatment and information on functional disabilities. However, for orthopedic surgeons filling out the form is extra work in addition to the information on diagnosis and treatment they provide to the referring physician, usually the general practitioner. Two orthopedic surgeons suggested to also giving a copy of this information to the occupational physician. This is not common practice right now and usually does not include information on functional limitations, while the occupational physicians appreciated this information on the form.

In this study the orthopedic surgeons had to add the procedure of filling out the form to their usual work, diagnosing and treating the patient. Since the form was only applicable for a small proportion of patients and will in most cases not change the treatment given by the orthopedic surgeon, implementing it into the routine of medical specialists will be difficult. In the interviews, the suggestion was given to let the occupational physicians take the initiative for information exchange; they need in the information in some cases in order to manage an employee's rehabilitation to work. Furthermore, according to the Gatekeeper Improvement Act, the occupational health service has to give an advice on the prognosis and the possibilities for reintegration for those employees on sick leave for six weeks and who will probably not return to work on short notice. At this moment the occupational physician needs the information as provided on the form, information on diagnosis and prognosis, in order to complete the advice.

The developed information exchange form does not leave room for specific questions regarding the disorder or the patient. Whether using a form on initiative of the occupational physician is more useful than a written request for information or providing the occupational physician with a copy of the letter send to the general practitioner cannot be answered in this study. Further research is needed to answer this question.

### Methodological considerations

Data triangulation was performed by means of interviewing both patients and physicians and by means of a member check: the interviewed physicians were asked whether the results as they are written down were a correct rendering of the information they provided. No medical files or other documentation was used; the data collected in the study was limited to the experience of the patients and physicians with the exchanged forms.

In this study it was decided that the orthopedic surgeon should use the information exchange form early in the treatment trajectory for all patients on sick leave with certain disorders. There was no difference between patients at risk for long term sick leave and patients who would only call sick for some days. However, when patients are on sick leave for only a few weeks, they might not visit their occupational physician. In this study that resulted in three forms not being transferred to the occupational physician. The suggestion to leave the initiative for using the form to the occupational physician might overcome this issue.

## Conclusion

The form provided occupational physicians with information on diagnosis, treatment, functional limitations, and prognosis of their patients treated by an orthopedic surgeon. According to the physicians and patients participating in this qualitative evaluation the form was useful and could be used in practice. Since the form was only used for a few patients, the question whether the form can be useful in the general practice cannot be answered satisfactorily. An important consideration for further exploration is whether usefulness of the application of the form is limited to the relatively small proportion of patients that do not recover as expected.

## Competing interests

The author(s) declare that they have no competing interests.

## Authors' contributions

EF, AB and AS developed the protocol for this study with assistance from HM and JV. EF developed the interview guide with help from AS, AB, HM and JV. EF recruited participants for the interviews, carried out the interviews and transcribed them. EF and AS analyzed the interviews. EF wrote the manuscript and revised it based on comments from AB, AS, HM and JV. All authors read and approved the final manuscript.

## Pre-publication history

The pre-publication history for this paper can be accessed here:



## Supplementary Material

Additional File 1 Communication Form for Knee Complaints. Click here for file

## Figures and Tables

**Table 1:** topic list for interviews

Patients	Is it correct that you visited your orthopedic surgeon on <date> because of your <knee/back> complaints?
	How is your <knee/back> now?
	If I am correct, your orthopedic surgeon filled out a form for your occupational physician, do you remember this?
	Did you receive this form?
	Did you give this form to your occupational physician?
	When and how did you give it/why did you not transfer it?
	What did the occupational physician do with this information?
	What did you think of the fact that your orthopedic surgeon informed your occupational physician in this way?
	Did your orthopedic surgeon contact your occupational physician?
	Do you have other questions or information on this topic?
Orthopedic surgeons and occupational physicians	Assessment of the form
	Usability of the form
	Impact of using the form on the management of the disorder
	Impact of the form on communication
	When is communication useful
