# Supplementary material for: Qualitative evaluation of a form for standardized information exchange between orthopedic surgeons and occupational physicians
Source: BMC Health Serv Res. 2006 Nov 2;6:144. doi: 10.1186/1472-6963-6-144 (PMC1635707; doi:10.1186/1472-6963-6-144)
Supplement: Additional File 1 — Communication Form for Knee Complaints. [file 1472-6963-6-144-S1.pdf]

Information from your orthopedic surgeon for your occupational physician
Knee disorders and work

Date……………………/……………………/200……

**Patient:**		**Orthopedic surgeon:**	
Name:	…………………………………………………	Name:	………………………………………
Date of birth:	…………../…………../19……..	Hospital:	………………………………………
		Place:	………………………………………
		Telephone:	………… - …………………………

Dear colleague,

Today I was consulted by your patient, who is currently working / on sickleave*

In order to start rehabilitation / to prevent sickleave* I would like to give you the following information:

(Provisional) diagnosis:

❑ Meniscal tear ❑ Ligament tear ❑ Arthrosis ❑ Patellar femorale syndrome

❑ Other/explanation* ……………………………………………………………………………………………

Proposed trajectory:

Additional diagnostics Expected date / Explanation

❑ MRI ………………………………………………………………………………………

❑ Other/explanation* ………………………………………………………………………………………

Therapy

❑ Expectative

❑ Conservative ❑ Rest during ………………………weeks

❑ Exercise therapy Physical therapy / Excercise therapy*

❑ Medication, viz. ………………………………………………………………………………

❑ Injection, viz. ………………………………………………………………………………

❑ Invasive, arthroscopic operation ………………………………………………………………………………

❑ Other/explanation* ……………………………………………………………………………………………

Current functional limitations, from medical perspective:

	not limited	limited	not allowed
Kneeling or squatting	❑	❑	❑
Walking stairs	❑	❑	❑
Sitting during workday	❑ ± 8 hrs	❑	❑
Standing during workday	❑ 6-8 hrs	❑	❑
Walking during workday	❑ 6-8 hrs	❑	❑

Other disabilities / explanation* ………………………………………………………………………………………

………………………………………………………………………………………

Regular change of posture ❑ wanted ❑ not necessary

Provisional prognosis:

I expect this patient to recover completely / almost completely / limited* within ……….. weeks

I expect that this patient can perform his usual activities after ……….. weeks

Next appointment with the patient: ❑ not ❑ within……….. weeks

Authorization:

…………………………………………………………………………………………(name patient)

hereby declares to give permission for exchanging the above information to his/her occupational physician. This permission regards only consultation necessary to gain sufficient information for adequate support during sick leave or for a rehabilitation plan and is only valid during the current episode of complaints. This information can only be used by the above mentioned physicians and the researchers. This information can not be distributed to third parties without my permission. The purpose of this information exchange is clear to me.

Signature of patient: ……………………………………………………………………………………………………

City: ………………………………………………………… Date …………………/…………………………/ 200……

* strike through what is not applicable.

This form is developed for the project "Employees at the Orthopedic Surgeon" Netherlands Expert Centre for Workrelated Musculoskeletal Disorders, march 2004
